# Comparison Between Moxifloxacin and Chloramphenicol for the Treatment of Bacterial Eye Infections

**DOI:** 10.1016/j.curtheres.2024.100740

**Published:** 2024-02-28

**Authors:** Valentina Gentili, Giovanni Strazzabosco, Rossella Spena, Sabrina Rizzo, Silvia Beltrami, Giovanna Schiuma, Andrea Alogna, Roberta Rizzo

**Affiliations:** 1 Department of Chemical, Pharmaceutical, and Agricultural Science, University of Ferrara, Ferrara, Italy; 2 Department of Translational Medicine, University of Ferrara, Ferrara, Italy

**Keywords:** Antibacterial activity, Antibiofilm, Cytotoxicity, Prophylaxis

## Abstract

**Background:**

Moxifloxacin is a bactericidal methoxyquinolone used for the treatment of conjunctivitis and prophylactic therapy in cataract and refractive surgeries. Chloramphenicol is a bacteriostatic organochlorine introduced into clinical practice in 1948 and used mainly in topical preparations because of its known toxicity.

**Objectives:**

The study aimed to evaluate the in vitro antibacterial effect and the ocular cytotoxicity of these broad-spectrum antibiotics.

**Methods:**

Antimicrobic activity was tested on 4 bacteria strains (*Escherichia coli, Pseudomonas aeruginosa, Staphylococcus aureus,* and *Staphylococcus epidermidis*), and determined through calculation of MIC and half inhibitory concentration for each microorganism. Antibacterial activity was determined by microdilution method after 24 hours’ incubation with 2-fold serial dilutions (2.5 mg/mL to 4.883 µg/mL) of moxifloxacin and chloramphenicol. Disk diffusion test were performed according to European Committee on Antimicrobial Susceptibility Testing methodology. Biofilm formation inhibition and biofilm eradication concentration assay were conducted for *P aeruginosa* and *S epidermidis* using the microdilution method. Cytotoxicity of antibiotics was evaluated by MTT (3-[4,5-dimethylthiazol-2-yl]-2,5 diphenyl tetrazolium bromide) colorimetric assay on human corneal cell.

**Results:**

Cytotoxicity of antibiotics was evaluated on human epithelial corneal cells after 4 hours treatment by viability assay. Results showed that corneal cell viability was significantly higher after moxifloxacin treatment compared with chloramphenicol (*P* < 0.01). Moxifloxacin is characterized by a significantly lower MIC and half inhibitory concentration values and a larger inhibition zone for all the strain tested, with high performance in controlling gram-negative growth, compared with chloramphenicol. Moreover, moxifloxacin showed higher activity compared with chloramphenicol in the inhibition of biofilm formation and in the disruption of biofilm, especially against *S epidermidis* biofilm.

**Conclusions:**

The lower corneal cell toxicity and the broader spectrum of antibacterial activity observed with moxifloxacin suggests its use in ophthalmic solution for the treatment of bacterial eye infections.

## Introduction

In the medical management of bacterial ocular infections, the treatment must be rapidly started, usually before pathogen identification. Therefore, the antibiotic of choice must be effective against a broad spectrum of potential ocular pathogens, providing good coverage against both gram-positive and gram-negative bacteria.^1^ Moreover, eye tolerability should also be taken into account to increase patients’ compliance and comfort.^2^ In this in vitro study, we compared the antibacterial activity of moxifloxacin and chloramphenicol on the bacteria strains that are the most common causes of ocular infections, as well as their tolerability on cultured human corneal cells.

Moxifloxacin is a relatively new fluoroquinolone with broad-spectrum antibacterial activity, acting by inhibition of DNA-girase and topoisomerase IV. The activity against bacterial but not mammalian essential enzymes enable fluoroquinolones to be selective and bactericidal.^3^^,^^4^ Ophthalmic preparations based on moxifloxacin are used in clinical practice for the treatment of conjunctivitis, keratitis, and as prophylactic therapy in refractive and cataract surgery.^5^^,^^6^

Due to its broad spectrum of activity and low cost, topical chloramphenicol has been utilized extensively in the treatment and prevention of superficial eye infections.^7^ Chloramphenicol has bacteriostatic activity by inhibiting selectively bacterial protein synthesis. It is used to treat conjunctivitis in ophthalmology general practice and for prophylaxis against infection during an ocular surgery. Many studies^8^^,^^9^ have been conducted on potential toxicity of chloramphenicol, even in cases of topical ocular administration. Most of the data affirm that topical chloramphenicol does not present significant toxicity and that it is generally well tolerated.^10^, ^11^, ^12^ Nonetheless, the risk of systematic complications due to absorption of the drug through the conjunctival and nasal mucosae should be considered.

## Materials and Methods

### Microbial strains and antimicrobic activity

Microorganism strains were used to test the antimicrobic activity of the 2 antibiotic solutions. For each strain, 2-fold serial dilution of antibiotics in sterile phosphate buffered saline (PBS) 1 × were used (ranging from 2.5 mg/mL to 0.038 µg/mL). The antibacterial effect was assessed against *Staphylococcus aureus* (ATCC 6538), *Staphylococcus epidermidis* (ATCC 12228), *Pseudomonas aeruginosa* (ATCC 9027), and *Escherichia coli* (ATCC 8739).

The MIC of moxifloxacin and chloramphenicol were determined by broth microdilution method in 96-well microplates (U bottom), and defined as the lowest concentration of drug that will inhibit visible growth of the organism after 24 hours’ incubation. An amount of 100 µL bacterial suspension were seeded with a final concentration of 5  ×  10^5^ CFU/mL in each well. After 24 hours of incubation at 37°C, MICs were assessed, and 50-µL samples from the wells at MIC, 2 × MIC, and 4 × MIC and from well with the highest antibiotic concentration (2.5 mg/mL) were plated on Mueller Hinton agar plates to determine the minimum bactericidal concentration (MBC) for each antibiotic and for each bacterial strain. After 24 hours’ incubation at 37°C, CFUs were counted. The MBC was defined as the lowest concentration of drug that resulted in ≥99.9% kill of the initial inoculum.^13^ MIC and MBC assays were performed in triplicate.

The half inhibitory concentration (IC_50_) was determined by broth microdilution method in 96-well microplates (flat bottom), using the same range of concentrations as for the MIC (from 2.5 mg/mL to 0.038 µg/mL). A microplate reader was used to measure the absorbance (ie, optical density) of the microplate wells at time 0, 1 hour, 2 hours, 4 hours, 6 hours, and after 24 hours of incubation. Means of absorbance measurements were calculated, graphed, and compared with untreated controls to determine the percent of microbial growth and IC_50_ was extrapolated for each species from the data obtained from the microdilution method by nonlinear regression.^14^ Microdilutions for IC_50_ calculation were performed in triplicate.

Disk diffusion tests were performed according to European Committee on Antimicrobial Susceptibility Testing methodology.^15^ The disks used contained moxifloxacin 5 µg and chloramphenicol 5 µg. The disks were placed on Mueller Hinton agar plates inoculated with bacteria at 0.5 McFarland immediately before disks positioning and incubated overnight at 37°C. The inhibition zone diameter was measured in millimeters. The data obtained are the results of 3 independent measurements.

### Biofilm formation inhibition and biofilm eradication concentration assays

Biofilm formation inhibition concentration (BIC) and biofilm eradication concentration (BEC) assays were conducted in 96-well plate for *P aeruginosa* and *S epidermidis* using the microdilution method.^16^ For the BIC assay, bacteria were seeded at 5 ×  10^5^ CFU/mL and treated with serial 2-fold dilutions of antibiotics (from 2.5 mg/mL to 0.038 µg/mL). The plates were then incubated at 37°C for 24 hours. Supernatants from each well were poured and planktonic cells were removed by washing with sterile PBS. The biofilm formation was stained using 1% crystal violet and incubated for 15 minutes at room temperature. Wells were then washed again with PBS to remove excess of dye that was finally solubilized with acetic acid 33%.^17^ The plate was read using a microtiter plate reader at 595 nm. For the BEC assay, each well was filled with 100 µL bacterial suspension at 5  ×  10^5^ CFU/mL and incubated for 24 hours at 37°C to allow biofilm formation. The day after, wells were carefully washed with PBS, avoiding biofilm detachment from well walls, and 150-µL antibiotic dilutions (from 5 mg/mL to 0.038 µg/mL) were added. Microplates were further incubated for 24 hours at 37 °C and then processed as the BIC assay. The percentage of BIC/BEC was calculated using the following formula: [(Absorbance growth control – Absorbance sample) / Absorbance growth control ] × 100. The biofilm assays results were obtained from 2 independent experiments, each performed in triplicate.

### Human cells and cytotoxicity assay

The human corneal epithelial cell line (HCE-2) (ATCC, CRL-11135) was cultured at 37°C and 5% carbon dioxide in keratinocyte serum-free medium (Gibco, Grand Island, New York) supplemented with 0.05 mg/mL bovine pituitary extract (Gibco), 5 ng/mL epidermal growth factor, 500 ng/mL hydrocortisone, and 0.005 mg/mL insulin (Gibco).

Cell viability of HCE-2 was evaluated after treatments using the colorimetric method MTT (3-[4,5-dimethylthiazole-2-yl]-2,5 diphenyltetrazolium bromide) (Roche, Basel, Switzerland), following the manufacturer protocol to measure the metabolic efficiency of living cells. Briefly, HCE-2 cells were seeded in 96-well plates at a density of 5 × 10^3^ cells per well and cultured overnight. The day after, HCE-2 cells were treated with commercial eyedrops at different 2-fold serial dilutions (ranging from 2.5 mg/mL to 0.078 mg/mL active compound) for 4 hours. After incubation time, MTT was performed and the absorbance measured at 450 nm using a microplate reader. The experiment was performed in triplicate.

### Statistical Analysis

Data were statistically compared using the Student *t* test. A value of *P* < 0.05 was considered statistically significant, and indicated with *P ≤ 0.05, **P ≤ 0.01, and ***P ≤ 0.001. Statistical analyses were performed using GraphPad Prism, version 8 (GraphPad Software, San Diego, California).

## Results

### Antibacterial activity

Antibacterial activity was preliminarily determined by microdilution method after 24 hours’ incubation. Antibiotics were 2-fold serially diluted (from 2.5 mg/mL to 0.038 µg/mL) in PBS 1 × and incubated with 5 × 10^6^ CFU/mL for each microorganism.

After 24 hours of treatment, absorbance was measured, and the values compared with the untreated bacteria (growth control). Table 1 shows the MIC value for moxifloxacin and chloramphenicol and for each bacterial strain. In particular, the activity of moxifloxacin against *E coli* and *S epidermidis*, presented a MIC value of 0.076 µg/mL because no bacterial growth was observed at this concentration. Moreover, the concentration needed to inhibit *S aureus* and *P aeruginosa* growth was significantly lower for moxifloxacin compared with chloramphenicol (19.53 vs 78.125 µg/mL and 0.61 µg/mL vs 39.063 µg/mL, respectively; *P* = 0.0001 by Student *t* test). The analysis of the subculture for the MBC confirmed that chloramphenicol has bacteriostatic activity for all the strains tested, whereas moxifloxacin revealed different behavior against gram-positive and gram-negative bacteria. Moxifloxacin MBC was 4-fold higher than the corresponding MIC for *E coli* and *P aeruginosa*, and corresponded to the MIC for *S aureus* and *S epidermidis*.

**Table 1:** MIC values after 24 hours of incubation. The experiment was performed in triplicate.

Bacteria strain	MIC, µg/mL*	
	Moxifloxacin	Chloramphenicol
Escherichia coli	0.076 (0.038–0.076)	19.53 (19.53)
Pseudomonas aeruginosa	0.61 (0.61–1.22)	39.063 (39.063–78.125)
Staphylococcus aureus	19.53 (19.53–39.063)	78.125 (78.125–156.25)
Staphylococcus epidermidis	0.076 (0.038–0.076)	78.125 (78.125)

⁎ Values are presented as MIC (95% CI).

Chloramphenicol zones of inhibition for *E coli, P aeruginosa, S aureus*, and *S epidermidis* were 28 mm (range = 27–28 mm), 16 mm (range = 15–16 mm), 20 mm (range = 20–20 mm) and 25 mm (range = 25–26 mm), respectively. Moxifloxacin zones of inhibition were higher for all bacteria tested, reaching 33 mm for *E coli* (range = 33–34 mm), 31 mm for *P aeruginosa* (range = 29–33 mm), 28 mm for *S aureus* (range = 26–29 mm), and *S epidermidis* (range = 27–28 mm) (see the Supplemental Figure in the online version at doi:10.1016/j.curtheres.2024.100740).

### IC_50_ determination

IC_50_ was calculated on the percentage of microorganism growth after 24 hours’ incubation in presence of 2-fold serial dilution of antibiotics (ranging from 2.5 mg/mL to 0.038 µg/mL). One hundred percent growth was determined in microorganisms without treatments. In Table 2, the IC_50_ values for each microorganism and for each antibiotic, expressed in micrograms per milliliter, are shown. The results demonstrated that moxifloxacin exhibited better inhibitory activity against all strains compared with those obtained by chloramphenicol because the concentration needed to inhibit bacterial proliferation was lowest. As a quinolone derivative, the best inhibitory activity of moxifloxacin was achieved against gram-negative bacteria, showing IC_50_ values of 0.000275 and 0.0003541 µg/mL for *E coli* and *P aeruginosa*, respectively.

**Table 2:** Half inhibitory concentration (IC_50_) values in %v/v. The experiment was performed in triplicate.

Bacteria strain	IC_50_, µg/mL*	
	Moxifloxacin	Chloramphenicol
Escherichia coli	0.000275 (0.000248–0.0003013)	2.666 (2.521–2.807)
Pseudomonas aeruginosa	0.0003541 (0.0003207–0.0003855)	9.783 (9.299–10.29)
Staphylococcus aureus	1.255 (1.033–1.488)	4.913 (4.432–5.356)
Staphylococcus epidermidis	0.9838 (0.6407–1.385)	4.311 (3.949–4.634)

⁎ Values are presented as IC_50_ (95% CI).

### BIC and BEC

Biofilm formation was influenced by both moxifloxacin and chloramphenicol. Chloramphenicol was able to inhibit the formation of *S epidermidis* and *P aeruginosa* biofilms at 78.125 and 39.063 µg/mL, respectively, with identical values as the respective MICs. The biofilm formation inhibition was reached by moxifloxacin at lower concentrations, in particular at 1.22 µg/mL for *P aeruginosa* biofilm and 0.15 µg/mL for *S epidermidis* biofilm, with values 2-fold higher than the respective MICs.

Complete biofilm eradication was not achieved with the 2 tested antibiotics even at the highest concentrations. Fifty percent of biofilm eradication was reached by chloramphenicol at 5 mg/mL (range = 2.5–5 mg/mL) and 1.25 mg/mL (range = 0.625–1.25 mg/mL), and by moxifloxacin at 5 mg/mL (range = 2.5–5 mg/mL) and 0.076 µg/mL (range = 0.038–0.076 µg/mL) against *P aeruginosa* and *S epidermidis* biofilms, respectively.

### Cytotoxicity on human corneal cells

The viability of corneal epithelial cells (HCE-2) after exposure to moxifloxacin and chloramphenicol is shown in the Figure. Antibiotics were added in cell medium at 2-fold serial dilutions (from 2.5 to 0.078 mg/mL) and incubated for 4 hours. Data of viability were confirmed by IC_50_ calculation, which showed a value of 1.443 mg/mL (95% CI, 1.102–1.911 mg/mL) for moxifloxacin, versus 0.6835 mg/mL (95% CI, 0.4618–1.017 mg/mL) for chloramphenicol. The results showed that corneal cell viability was significantly higher after moxifloxacin treatment compared with chloramphenicol at all concentrations tested.

**Fig:**
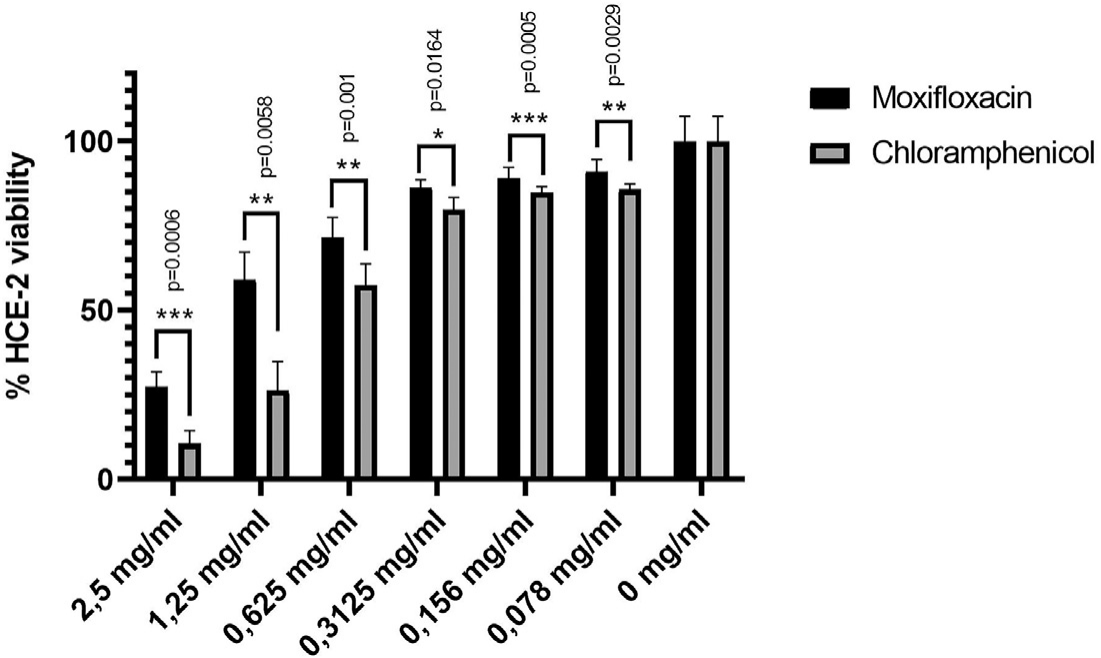
Evaluation of MTT assay in the human corneal epithelial cell line (HCE-2) after 4 hours of incubation with moxifloxacin and chloramphenicol. Data are expressed as percentage of the maximum cell viability; mean ± SE of at least 3 experiments performed in triplicate.

## Discussion

Our study comparatively evaluated the in vitro effects of 2 antibiotics commonly formulated in eye drops for the treatment of bacterial infections and pre- and postoperative prophylaxis. Moxifloxacin is a broad spectrum, 8-methoxy fluoroquinolone with bactericidal, concentration dependent, antibacterial activity. Chloramphenicol is a broad-spectrum antibiotic with bacteriostatic action, confirmed by the MBC analysis. Both antibiotics are considered broad spectrum, but moxifloxacin is characterized by a higher efficiency in counteract gram-negative bacterial infection in comparison with chloramphenicol. Indeed, we observed significantly lower MIC and IC_50_ values for moxifloxacin for all the strain tested, as well as a larger inhibition zone for all tested bacteria compared with chloramphenicol. Because the presence of bacterial biofilms has been demonstrated also in ocular infections, we evaluated the ability of chloramphenicol and moxifloxacin to inhibit the formation or disrupt the presence of biofilms. Again, moxifloxacin showed higher activity compared with chloramphenicol in the inhibition of biofilm formation and in the disruption of biofilm, especially against *S epidermidis* biofilm.

Along with the antibacterial activity, the tolerability profile needs to be considered because the effects on viability of corneal epithelial cells are critical in the selection of a topical antimicrobial solution. Local toxic effects of ophthalmic formulations may be masked by manifestations of the treated ocular disease and irritation caused by a drug is difficult to distinguish from the disease itself.^18^ To assess the cytotoxicity of moxifloxacin and chloramphenicol, we compared the viability of HCE-2 cells after in vitro treatment. The results demonstrate a significant influence of chloramphenicol on human cell viability for the same concentrations used for moxifloxacin. The lower corneal cell toxicity observed in the presence of moxifloxacin suggests its safe use in ophthalmic solution for the treatment of bacterial eye infections.

## Conclusions

The choice of an antibiotic for ocular infections often considers only its efficiency against a broad spectrum of potential ocular pathogens, but also eye tolerability should be taken into account to increase patients’ compliance and comfort. Assessment of in vitro toxicity, together with the antibacterial potential, might help clinicians in the selection and use of available drugs to obtain a targeted therapy.

## Declaration of competing interest

The authors have indicated that they have no conflicts of interest regarding the content of this article.

## Appendix Supplementary materials

**Supplemental Fig.** Representative pictures of inhibition zones of chloramphenicol (F) and moxifloxacin (M) 5 µg-disks against *Escherichia coli* (coli), *Pseudomonas aeruginosa* (aer), *Staphylococcus aureus* (aureus), and *Staphylococcus epidermidis* (epidermidis) seeded in 90-mm plates of Mueller-Hinton agar. Image, image 1
