# Supplementary figures and images for: Comparison Between Moxifloxacin and Chloramphenicol for the Treatment of Bacterial Eye Infections
Source: Curr Ther Res Clin Exp. 2024 Feb 28;100:100740. doi: 10.1016/j.curtheres.2024.100740 (PMC10950741; doi:10.1016/j.curtheres.2024.100740)

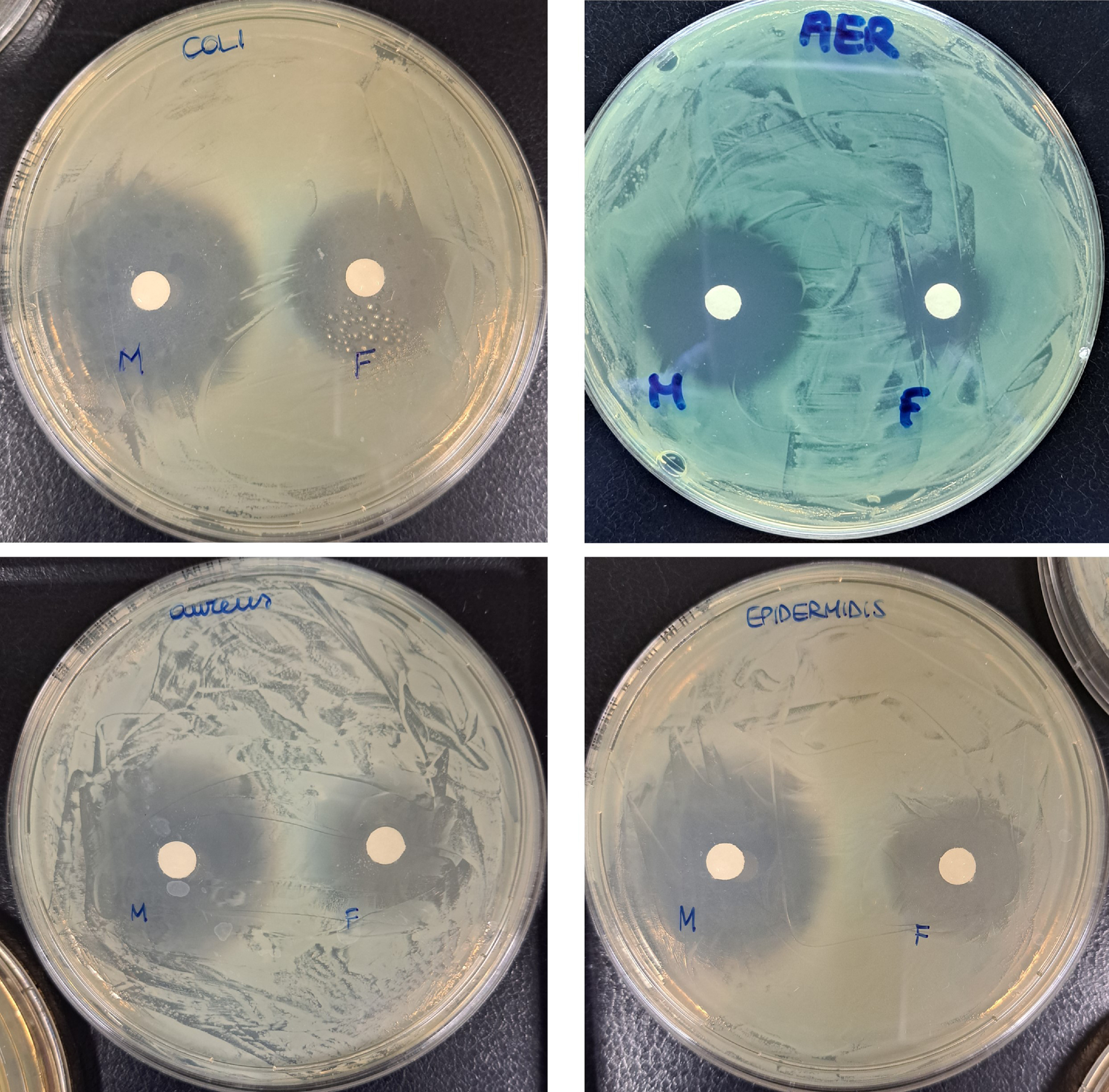

Supplement: Supplementary file 1 — Supplemental Fig. Representative pictures of inhibition zones of chloramphenicol (F) and moxifloxacin (M) 5 µg-disks against Escherichia coli (coli), Pseudomonas aeruginosa (aer), Staphylococcus aureus (aureus), and Staphylococcus epidermidis (epidermidis) seeded in 90-mm plates of Mueller-Hinton agar. [file mmc1.jpg]
